# Modified Transabdominal Oocyte Retrieval Guided by Vaginal Ultrasound Probe: A Case Report and Literature Review

**DOI:** 10.1155/crog/5530041

**Published:** 2025-11-17

**Authors:** Gang Yang, Shengran Wang, Haiyan Lin, Ping Yuan, Qingxue Zhang, Hui Chen

**Affiliations:** ^1^Center for Reproductive Medicine, Sun Yat-sen Memorial Hospital, Sun Yat-sen University, Guangzhou, China; ^2^Center for Reproductive Medicine, Shenshan Medical Center, Sun Yat-sen Memorial Hospital, Sun Yat-sen University, Shanwei, China; ^3^Guangdong Provincial Clinical Research Center for Obstetrical and Gynecological Diseases, Guangzhou, China

**Keywords:** case report, ectopic ovary, in vitro fertilization, transabdominal oocyte retrieval, ultrasound probe

## Abstract

For patients with ectopic ovaries undergoing in vitro fertilization-embryo transfer, transabdominal ovarian puncture remains necessary for selected cases. Despite advancements in the procedure, transabdominal oocyte retrieval demands higher operational skills compared to transvaginal retrieval. In this study, a retrospective analysis was conducted on the clinical data and treatment process of a patient with a unilateral ectopic ovary, undetectable by vaginal ultrasound, who underwent transabdominal ovarian puncture for oocyte retrieval under the guidance of a vaginal ultrasound probe. By employing a towel clip to grasp and indent the abdominal skin, simulating a vaginal fornix-like structure, the vaginal ultrasound probe was flexibly positioned on the smooth abdominal wall, facilitating the oocyte retrieval process and ensuring its smooth execution. A significant number of oocytes were successfully retrieved, resulting in a successful pregnancy and term live birth. This modified technique, which further optimizes the operational process, is an efficient and reliable method for transabdominal oocyte retrieval.

## 1. Introduction

Oocyte retrieval is a critical component of in vitro fertilization-embryo transfer (IVF-ET). Traditionally, ultrasound-guided transvaginal ovarian puncture is the standard clinical approach for oocyte retrieval [1]. However, in some instances involving infertility patients with ectopic ovaries—those undetectable by transvaginal ultrasound due to congenital reproductive system malformations, postoperative pelvic-abdominal adhesions, or ovarian transposition surgery—ultrasound-guided transabdominal ovarian puncture may be necessary. This approach is particularly relevant when the ectopic ovary is located close to the abdominal wall and exhibits limited mobility during IVF treatment [2–5]. This study presents a case of successful pregnancy following modified transabdominal oocyte retrieval guided by a vaginal ultrasound probe. A comprehensive literature review is conducted to discuss the operational techniques and precautions associated with this method, with the goal of optimizing the procedure for ectopic ovarian puncture and oocyte retrieval.

## 2. Case Presentation

A 36-year-old female patient presented with a history of infertility following a natural abortion in 2020 and sought assisted reproductive treatment at our center. The patient, who married in 2013, engaged in regular sexual activity and used condoms for contraception until 2016. Hysterosalpingography performed at our hospital in March 2019 revealed that the left fallopian tube was patent, while the right fallopian tube was obstructed. In March 2020, the patient experienced a spontaneous abortion at 8 weeks of gestation following a natural pregnancy. Subsequently, in April 2020, she underwent hysteroscopic adhesiolysis for uterine adhesions and removal of retained products of conception (RPOC). A follow-up hysteroscopy in July 2020 demonstrated a nearly normal uterine cavity. Despite discontinuing contraception, the patient was unable to conceive and thus sought fertility treatment (Table 1).

**Figure 1:**
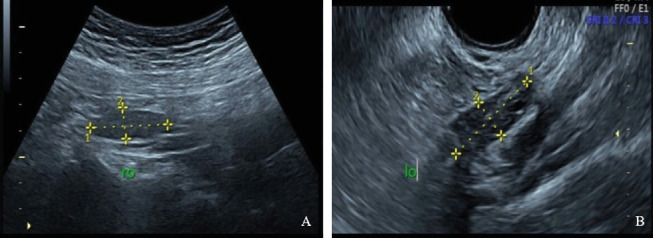
Combined transabdominal and transvaginal three-dimensional Doppler ultrasonography examination. (A) The right ovary is only detectable via transabdominal ultrasound. (B) The left ovary is visible through transvaginal ultrasound.

The patient reported no history of hypertension, diabetes, heart disease, or significant family medical conditions. Menarche occurred at age 12, with irregular menstrual cycles of 5–6 days every 2–4 months and normal menstrual volume without dysmenorrhea. Obstetric history was recorded as G1P0A1E0. The body mass index (BMI) was 25.0 kg/m^2^, with no remarkable abnormalities detected in general physical or pelvic examinations. Imaging studies, including transabdominal and transvaginal three-dimensional Doppler ultrasonography, revealed that the right ovary was positioned higher and closer to the abdominal wall, undetectable by transvaginal ultrasound. In contrast, the left ovary was in a normal position (Figure 1). The antral follicle count (AFC) was seven follicles in each ovary. Sexual hormonal assays showed the following: anti-Müllerian hormone (AMH) 3.73 ng/mL, follicle-stimulating hormone (FSH) 8.20 U/L, and luteinizing hormone (LH) 9.14 U/L. The chromosomal karyotype is 46, XX. The admission diagnosis included secondary infertility, ovulatory dysfunction, right fallopian tube obstruction, right ectopic ovary, post-intrauterine adhesion release surgery, and abnormal pregnancy history. The male partner had no significant medical history, with semen analysis or physical examination showing no apparent abnormalities; his chromosomal karyotype is 46, XY. Given the strong desire of the couple to conceive, assisted reproductive technology treatment was indicated, leading to a comprehensive evaluation and subsequent IVF treatment.

In response to the specific situation of the patient, a long-acting GnRH agonist protocol was employed for controlled ovarian hyperstimulation (COH). During menstruation, the patient was administered 3.75 mg of triptorelin acetate (Diphereline, Ipsen, France) for downregulation. Four weeks later, gonadotropin (Gn) urofollitropin (Lishenbao, Livzon, China) was initiated to promote follicular growth, with a daily intramuscular dose of 150 U. Follicular development was monitored using transvaginal ultrasound for the left ovary and transabdominal ultrasound for the right ovary, with adjustments to Gn dosage made based on follicular growth and hormonal levels. Gn was administered for a total of 16 days, with a cumulative dosage of 3075 U. When more than three dominant follicles reached a diameter of ≥ 17 mm, and serum E2 levels reached 2682.00 pg/mL (9842.94 pmol/L), 8000 U of human chorionic gonadotropin (HCG) (choriogonadotropin, Livzon, China) was administered intramuscularly to trigger ovulation. Oocyte retrieval was performed 36 h later. Given the absence of significant developmental differences between the ovarian follicles and the patient's preference to retrieve from both ovaries, a combined approach of transvaginal puncture of the left ovary and transabdominal puncture of the right ovary was decided upon after informed consent and ethical approval.

**Figure 2:**
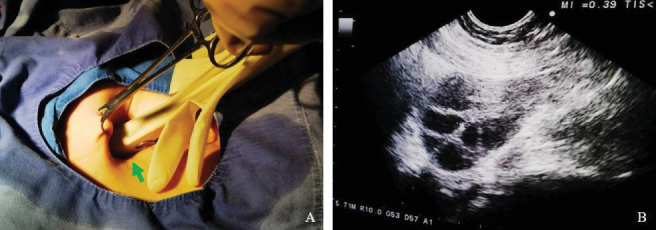
Transabdominal detection of the right ovary using a vaginal ultrasound probe. (A) Ultrasound probe positioned within the abdominal skin fold. (B) Clear ultrasound image of the right ovary.

**Figure 3:**
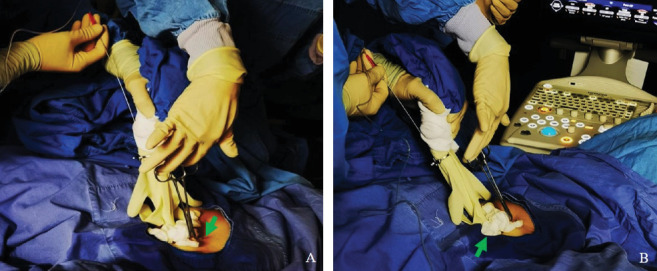
Modified transabdominal ovarian puncture for oocyte retrieval procedure. (A) Surgical towel clip elevating the abdominal skin to create a vaginal fornix-like concave structure. (B) Sterile moist gauze wrapped around the ultrasound probe and skin concave.

The patient was positioned in lithotomy for the procedure. Conventional transvaginal puncture of the left ovary was performed under vaginal ultrasound guidance, resulting in the puncture of seven dominant follicles and retrieval of five oocytes, achieving a retrieval rate of 71.4%. Following this, the patient remained in the lithotomy position, provided there was no reactive bleeding at the vaginal puncture site, while a vaginal ultrasound probe was used to locate the right ovary via the abdomen. Imaging confirmed the right ovary was approximately 40 mm to the right of the umbilicus, near the inner edge of the abdominal wall, about 35 mm from the outer edge (Figure 2). For the transabdominal oocyte retrieval, the abdominal skin was disinfected, draped, and wiped with physiological saline. A surgical assistant created a skin fold near the puncture site to mimic the vaginal fornix using surgical cloth forceps. A 7.5-MHz vaginal ultrasound probe (Aloka, Japan) with a puncture stent was then inserted into the skin fold. The probe tip and the skin concave were covered with sterile gauze soaked in saline, and its direction and angle were adjusted to visualize the right ovary. A 17-G single-lumen needle (Wallace, United States) was inserted through the probe stent, penetrating the skin and subcutaneous tissue to reach the right ovary. Each dominant follicle was aspirated sequentially along the same guiding line (Figure 3). The right ovary exhibited limited mobility and low resistance, facilitating the puncture of seven dominant follicles and retrieval of six oocytes, achieving an oocyte retrieval rate of 85.7%. The entire procedure was performed under intravenous general anesthesia, lasting approximately 30 min. The surgery proceeded without complications, with no bleeding at the puncture site or fluid accumulation in the pelvic and abdominal cavities, as confirmed by transvaginal–transabdominal combined Doppler ultrasound examination. Postoperative recovery was uneventful, with no symptoms of fever or abdominal pain. Out of the 11 oocytes retrieved and subjected to IVF, only one was normally fertilized. Two cleavage-stage embryos were transferred, but the cycle did not result in pregnancy.

In the second cycle, a GnRH antagonist protocol was adopted, with COH conducted according to standard procedures. The oocyte retrieval process mirrored that of the first cycle. On the trigger day, serum hormone levels indicated E2 >5000.00 pg/mL (18,350.00 pmol/L). A total of nine dominant follicles were punctured in the left ovary, resulting in the retrieval of eight oocytes, achieving an oocyte retrieval rate of 88.9%. Similarly, nine dominant follicles were punctured in the right ovary, yielding eight oocytes, also with an oocyte retrieval rate of 88.9%. The procedure proceeded smoothly, with stable vital signs throughout the operation. Postoperatively, there was no bleeding at the puncture sites or in the pelvic-abdominal cavity, and the patient was safely transferred to the recovery room. Given the low fertilization rate observed in the first cycle, intracytoplasmic sperm injection (ICSI) was utilized. Of the 16 oocytes retrieved, 15 were mature, and 12 underwent normal fertilization. Fresh embryo transfer was canceled to mitigate the risk of ovarian hyperstimulation syndrome (OHSS). Consequently, two cleavage-stage embryos and three blastocysts were cryopreserved.

For endometrial preparation prior to frozen embryo transfer (FET), a hormone replacement therapy (HRT) was implemented. On the fourth day of the first menstrual cycle following oocyte retrieval, 3.75 mg of triptorelin acetate (Diphereline, Ipsen, France) was administered for downregulation. Four weeks later, 2 mg of oral estradiol valerate tablets (Progynova, Bayer, France) were started twice daily, with the dose increased to 3 mg twice daily after 5 days. On the 12th day of estrogen administration, the endometrial thickness reached 8 mm with a clear triple-line sign. Progesterone support was then initiated with micronized progesterone capsules (Utrogestan, Bayer, France) 400 mg for vaginal administration twice daily and dydrogesterone tablets (Duphaston, Bayer, France) 20 mg orally twice daily to transform the endometrium. Prior to the transfer, 20 *μ*g of recombinant HCG (Ovidrel, Merck, Italy) was infused into the uterine cavity. Two thawed blastocysts were transferred, and luteal support was continued as previously described. Two weeks after embryo transfer, *β*-HCG levels were positive, and a Doppler ultrasound performed 4 weeks later confirmed an intrauterine dichorionic twin pregnancy. Regular prenatal examinations were conducted throughout the pregnancy. One fetus developed without noticeable abnormalities, while the other fetus experienced demise in the first trimester. At 38 weeks and 2 days of pregnancy, a healthy male infant was delivered via cesarean section, weighing 2950 g with no deformities. At 3 years and 3 months old, the child remains in good health.

## 3. Discussion

This research reports the successful implementation of transabdominal oocyte retrieval guided by a vaginal ultrasound probe, utilizing a simplified and flexible approach with an artificially created depression in the abdominal skin. The procedure was completed smoothly, achieving a high rate of oocyte recovery without significant postoperative complications such as pelvic or abdominal bleeding, abdominal pain, or infection. Following the transfer of thawed blastocysts, the patient conceived successfully and delivered a healthy newborn via cesarean section at full term, with no congenital disabilities or perinatal complications. This case represents the second instance of combined transvaginal and transabdominal oocyte retrieval performed at our center.

Oocyte retrieval techniques in IVF have evolved from invasive procedures, such as laparotomy and laparoscopy, to minimally invasive methods like transabdominal-transvesical puncture [6]. Ultrasound-guided transvaginal ovarian puncture is now widely recognized as the most straightforward and least invasive method, becoming routine practice in major reproductive centers. However, for specific IVF patients with ectopic ovaries—due to either congenital or acquired conditions—where ovaries are not detectable by transvaginal ultrasound, alternative methods may be required. In such cases, a combination of techniques may be considered after a thorough evaluation of the benefits and risks and obtaining informed consent, with transabdominal ovarian puncture remaining necessary for selected cases. Recent literature has reported the use of both abdominal [7, 8] and vaginal [9–11] ultrasound probes for guidance in transabdominal oocyte retrieval (Table 2). Despite advancements in the procedure, transabdominal oocyte retrieval demands higher operational skills compared to transvaginal retrieval [12]. Unlike previous reports, this study utilized a vaginal ultrasound probe inserted into a concave created in the abdominal skin by a towel clip, allowing for flexible and timely adjustments of the probe's direction and angle. This approach facilitated the puncture and retrieval process, offering several advantages, including prevention of probe slippage, enhanced ultrasound visualization, precise localization, and adaptable directional control. This modified technique has further optimized the operational process of transabdominal oocyte retrieval.

This modified procedure exhibits several notable characteristics. Firstly, while surgeons are well acquainted with the flexibility and precise directionality provided by the vaginal ultrasound probe in standard transvaginal ovarian puncture, the use of this probe in transabdominal oocyte retrieval minimizes the discomfort and complexity associated with abdominal ultrasound probe guidance. This approach eliminates the need for additional abdominal ultrasound probes and their associated puncturing instruments. Secondly, by using a towel clip to grasp and lift the abdominal skin, a depression resembling the vaginal fornix is created. Inserting the vaginal ultrasound probe into this concave area allows the operator to achieve a surgical sensation akin to that of conventional transvaginal ovarian puncture, making the surgical process more intuitive and avoiding noticeable scars on the abdominal wall. Additionally, wrapping the vaginal ultrasound probe with moist sterile gauze and using sterile saline solution, rather than an ultrasound coupling agent, prevents excessive probe movement while ensuring a clear ultrasound image. This modification effectively facilitates the smooth execution of the oocyte retrieval procedure.

Transabdominal puncture for oocyte retrieval is indicated when a unilateral or bilateral ovary is positioned close to the abdominal wall and is difficult to detect via transvaginal ultrasound. However, potential complications including anesthetic risks, visceral injury, and postoperative infection must be considered. Caution is warranted if the ectopic ovary exhibits excessive mobility (due to intense diaphragmatic breathing), is located too deep from the abdominal wall (as in cases of overweight or obesity), or is in close proximity to vital organs (such as intestines or aberrant large blood vessels). Comprehensive evaluation and stringent monitoring are essential during the procedure. If a substantial number of oocytes can be obtained from a normally positioned ovary via transvaginal puncture, it may be advisable to forgo puncturing the ectopic ovary. If puncturing the ectopic ovary is deemed necessary and transabdominal oocyte retrieval is unsuitable after thorough assessment, laparoscopic oocyte retrieval may be considered as an alternative [13]. Transabdominal oocyte retrieval is significantly affected by diaphragmatic breathing. Therefore, it is crucial to ensure airway patency and to carefully control the depth of anesthesia to minimize the adverse effects caused by ovarian displacement due to respiratory movements.

The current study is subject to several limitations. Most notably, its single-case design inherently restricts the generalizability of the findings. Larger studies are warranted to validate the reproducibility and broader applicability of this technique. Additionally, successful execution of the procedure requires substantial expertise in both vaginal ultrasound probe guidance and transabdominal operative techniques, underscoring the necessity for specialized training prior to its adoption in clinical practice.

## 4. Patient Perspective

The patient reported satisfaction both with the treatment process and its final outcome. With three cryopreserved embryos remaining, she expresses the intention to undergo a subsequent FET to achieve a second pregnancy in the future.

## 5. Conclusion

For infertility patients undergoing IVF treatment, when the ectopic ovary is close to the abdominal wall and exhibits limited mobility, a modified transabdominal oocyte retrieval guided by a vaginal ultrasound probe may be employed. This method is straightforward, reliable, and facilitates an increased number of oocytes collected, thereby improving the cumulative pregnancy rate and enhancing pregnancy outcomes. Additionally, this modified technique provides a valuable reference for surgeons performing various percutaneous puncture operations under ultrasound guidance, potentially benefiting a broader patient population.

## Figures and Tables

**Table 1:** Diagnostic and treatment timeline prior to IVF intervention.

**Date**	**Key event**	**Diagnostic/intervention**
Mar 2019	Hysterosalpingography	Unilateral tubal occlusion
Mar 2020	Spontaneous abortion	Expectant management
Apr 2020	Hysteroscopic surgery	Intrauterine adhesiolysis
Jul 2020	Follow-up hysteroscopy	Normal uterine cavity

**Table 2:** Summary and comparison of transabdominal oocyte retrieval techniques.

**Study (year)**	**Patient characteristics**	**Probe type**	**Special technique**	**Outcome**
Barton et al. (2011) [7]	Ectopic ovary	Abdominal	None	Tends to slip
Pereira et al. (2021) [8]	Ectopic ovary	Abdominal	None	Tends to slip
Sonmezer et al. (2021) [9]	Ectopic ovary or virginity	Vaginal	None	Tends to slip
Jia et al. (2023) [10]	Ectopic ovary	Vaginal	None	Tends to slip
Sonmezer et al. (2023) [11]	Virginity	Vaginal	None	Tends to slip
The present study	Ectopic ovary	Vaginal	Artificial skin fold	No slippage

## Data Availability

Data sharing is not applicable to this article as no new data were created or analyzed in this study.

## Supporting Information

Supporting Information Additional supporting information can be found online in the Supporting Information section. Supporting Information 1: The formation and function of artificial abdominal skin fold.
