# Supplementary figures and images for: Modified Transabdominal Oocyte Retrieval Guided by Vaginal Ultrasound Probe: A Case Report and Literature Review
Source: Case Rep Obstet Gynecol. 2025 Nov 17;2025:5530041. doi: 10.1155/crog/5530041 (PMC12643683; doi:10.1155/crog/5530041)

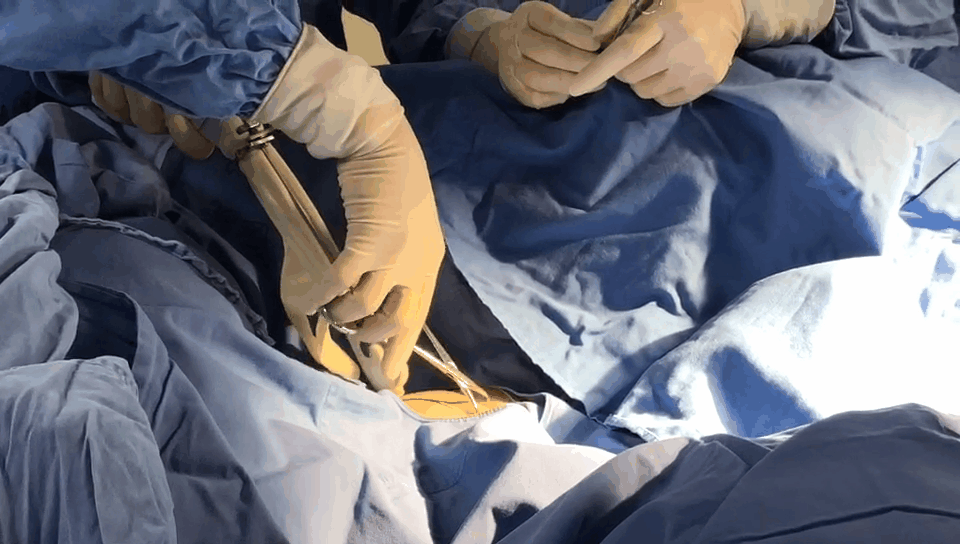

Supplement: Supporting Information — Additional supporting information can be found online in the Supporting Information section. Supporting Information 1: The formation and function of artificial abdominal skin fold. [file 5530041.f1.gif]
